# The pseudo-single-crystal method: a third approach to crystal structure determination

**DOI:** 10.1107/S0021889809013430

**Published:** 2009-05-15

**Authors:** Tsunehisa Kimura, Chengkang Chang, Fumiko Kimura, Masataka Maeyama

**Affiliations:** a Graduate School of Agriculture, Kyoto University, Kitashirakawa, Sakyo-ku, Kyoto 606-8502, Japan; b Tsukuba Magnet Laboratory, National Institute for Materials Science, 3-13 Sakura, Tsukuba, Ibaraki 305-0003, Japan; c Rigaku Corporation, 3-9-12 Matsubara-cho, Akishima, Tokyo 196-8666, Japan

**Keywords:** pseudo single crystals, X-ray diffraction, LiCoPO_4_ microrods

## Abstract

A novel method that enables single-crystal diffraction data to be obtained from a powder sample is presented. A suspension of LiCoPO_4_ microrods was subjected to a frequency-modulated dynamic elliptical magnetic field to align the microrods; the alignment achieved was consolidated by photopolymerization of the suspending UV-curable monomer. The composite thus obtained (referred to as a pseudo single crystal) gave rise to X-ray diffraction data from which the crystal structure was solved using the standard method for single-crystal X-ray analyses. The structure determined was in good agreement with that reported using a conventional single crystal.

## Abstract 2

A novel method that enables single-crystal diffraction data to be obtained from a powder sample is presented.

## 1. Introduction

Preparation of a single crystal is the primary key to a successful X-ray analysis. However, it is often difficult to prepare a single crystal large enough for X-ray measurement because of the difficulties in crystal growth arising from the complicated structures of compounds developed in advanced pharmaceutical and materials sciences. Furthermore, the demand for crystallographic analysis of nano- and microcrystals is increasing; however, their size is unsuitable for traditional single-crystal X-ray analysis. Sample size limits are even more severe for neutron diffraction measurements. Instead of the single-crystal method, an *ab initio* or direct-space method is now available for the analysis of powder diffraction (David *et al.*, 2002 ▶; Harris & Cheung, 2004 ▶). However, the use of this method is limited to specialists who can correctly determine the cell constants and structure from one-dimensional information alone. Preferential orientation of a powder sample is utilized to produce single-crystal-like diffraction data (Wessels *et al.*, 1999 ▶). The data quality strongly depends on the quality of the orientation.

Feeble magnetic materials such as most biological, organic, polymeric and inorganic materials respond to applied magnetic fields, albeit weakly. A number of studies concerning magnetic alignment of such materials have been reported (Maret & Dransfeld, 1985 ▶; Yamaguchi & Tanimoto, 2006 ▶). Biaxial crystal systems, *i.e.* the orthorhombic, monoclinic and triclinic systems, are magnetically characterized by three different magnetic susceptibilities (Nye, 1985 ▶). Using this magnetic nature, it is possible to align the crystal three-dimensionally with a specially designed dynamic magnetic field (Kimura & Yoshino, 2005 ▶). This technique has been proven to be useful in the alignment of high-*T*_c_ superconducting materials (Staines, 1996 ▶) and in crystallographic studies (Kimura *et al.*, 2006 ▶) where powder crystallites are converted into a pseudo single crystal (PSC: a solid composite in which crystallites are three-dimensionally oriented). The crystallite size *V* required for this technique is governed by the competition between the thermal and anisotropic magnetic energies and expressed by
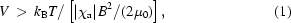
where *μ*_0_ is the magnetic permeability of a vacuum, *B* is the magnetic flux density, *χ*_a_ is the anisotropic magnetic susceptibility, *k*_B_ is the Boltzmann constant and *T* is the temperature. Typically, the size necessary is about the order of micrometres, depending on the field strength employed and the magnetic anisotropy of the crystal.

In a previous paper (Kimura *et al.*, 2006 ▶), it was demonstrated that l-alanine powder crystallites were converted to a PSC that produced well resolved X-ray diffraction spots. In this study, lithium cobalt phosphate (LiCoPO_4_) microrods were used to demonstrate that the crystal structure can be solved using X-ray diffraction from a PSC prepared from a powder sample. Because of the paramagnetic nature of this material and the resultant high magnetic anisotropy (Creer & Troup, 1970 ▶), only a moderate magnetic field (0.3 T) was needed to achieve three-dimensional alignment. The diffraction data were analysed by the standard method for single-crystal X-ray analyses. The resulting crystal structure was in excellent agreement with that reported for the single crystal (Kubel, 1994 ▶).

## 2. Experimental

The powder of LiCoPO_4_ was prepared *via* a modified hydrothermal method (Huang *et al.*, 2005 ▶). All the starting materials were of analytical grade and used without further purification. In a typical routine, Co(NO_3_)_2_·6H_2_O (0.01 mol) and H_3_PO_4_ (0.01 mol) were dissolved in 10 ml of distilled water separately and then mixed together to obtain a clear solution. LiOH solution (20 ml), prepared by dissolving 0.02 mol of LiOH·H_2_O in 20 ml of distilled water, was added dropwise to the above clear solution with continuous stirring. A bright blue suspension was obtained immediately after all the LiOH solution was added. The pH value was then adjusted to 8.5 by adding concentrated aqueous ammonia solution under vigorous stirring. The suspension was supersonically treated for 30 min before it was sealed into a 50 ml Teflon-lined autoclave and heat treated in an electric oven at 493 K for 16 h. After the hydrothermal treatment, the autoclave was cooled naturally, and a bright-red–purple powder was collected and washed several times with distilled water. The final powder was then dried at 333 K for 15 h.

**Figure 1:**
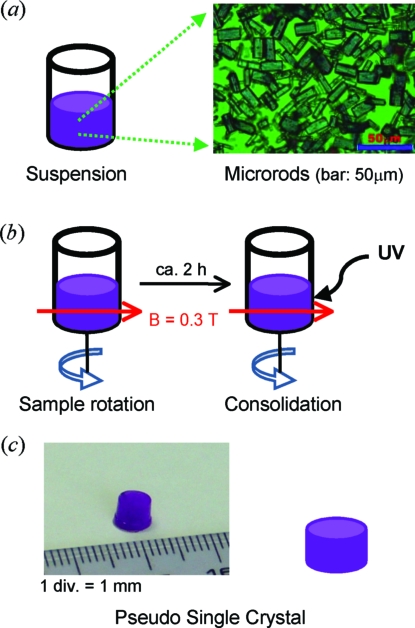
Scheme for the preparation of a pseudo single crystal (PSC) from LiCoPO_4_ microrods. (*a*) The microrods are suspended in a UV-curable monomer, then (*b*) the suspension is non-uniformly rotated in a static magnetic field to achieve a three-dimensional orientation, followed by consolidation of the matrix monomer by UV irradiation to obtain (*c*) a PSC.

Fig. 1 ▶ shows a flowchart of the preparation of a PSC. A micrograph of the synthesized microrods is shown in Fig. 1 ▶(*a*). The size of each rod is approximately 20 µm. This powder was mixed with a UV-curable monomer (Kyoritsu Chemical Co. Ltd, No. 8815; viscosity 1.2 Pa s) to prepare a suspension. The concentration of crystallites in the monomer was *ca* 10%(*v*/*v*). A small amount of the suspension was poured into a plastic container (diameter 5 mm, height 10 mm) and subjected to a frequency-modulated dynamic elliptical magnetic field (Fig. 1 ▶ *b*) to achieve three-dimensional alignment. After *ca* 2 h, the sample was irradiated with UV light to polymerize the monomer, and the alignment was fixed to obtain an oriented crystallites/polymer composite, the PSC (Fig. 1 ▶ *c*).

**Figure 2:**
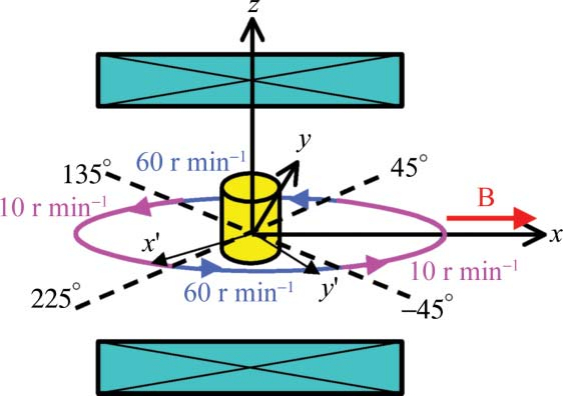
Schematic diagram showing the non-uniform sample rotation in a static magnetic field *B* (0.3 T). The magnetic field is parallel to the laboratory *x* axis and the rotation axis is parallel to the laboratory *z* axis. Rotation speed is switched at every 90°. The *x*′ and *y*′ axes are embedded in the sample.

A frequency-modulated dynamic elliptical magnetic field was generated by sample rotation in a static magnetic field, instead of rotation of the magnetic field. The rotating method is shown schematically in Fig. 2 ▶. The plastic container containing the suspension was mounted on a rotating unit, which was placed in the bore centre of a JASTEC cryogen-free superconducting magnet, generating a uniform 0.3 T horizontal magnetic field. The direction of the field was along the *x* axis (horizontal) of the laboratory frame. The rotating unit was revolved non-uniformly with its rotation axis along the vertical (parallel to the *z* axis), which has an effect that is equivalent to applying the dynamic elliptical magnetic field.

Two different rotation speeds, 10 and 60 r min^−1^, were employed, and the sample rotation was performed so that the rotation speed was switched at every 90° in a manner such as 10 r min^−1^ (−45 to 45°) 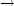 60 r min^−1^ (45 to 135°) 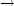 10 r min^−1^ (135 to 225°) 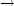 60 r min^−1^ (225 to −45°). Here, the 0 and 180° directions (parallel to the *x* axis) coincide with the field direction. The rotation speed was 10 r min^−1^ when the *x*′ axis, which is embedded in the rotating unit, passed around the *x* axis and it was 60 r min^−1^ when the *x*′ axis passed around the *y* axis. As a result, the *x*′ axis was exposed to the magnetic field for a longer time. With this dynamic magnetic field, the easy magnetization axes of the crystallites align in the *x*′ direction, and their hard magnetization axes align parallel to the rotating axis (the *z* direction), resulting in the three-dimensional (biaxial) alignment.

The PSC obtained (diameter 5 mm, height *ca* 5 mm) is shown in Fig. 1 ▶(*c*). A violet, platelet-shaped specimen with approximate dimensions of 3.00 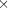 3.00 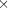 0.30 mm was cut from this composite and mounted on a glass fibre for X-ray measurement. All measurements were performed on a Rigaku R-AXIS RAPID diffractometer equipped with an imaging-plate area detector using graphite-monochromated Mo *K*α radiation. A total of 96 images that were exposed for 60 s/° were collected. The crystal-to-detector distance was 127.4 mm.

## 3. Results and discussion

**Figure 3:**
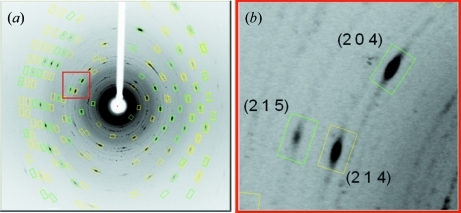
(*a*) A typical X-ray diffraction image (oscillation angle 5°) of a pseudo single crystal of LiCoPO_4_ is shown with diffraction spots enclosed by prediction rectangles. (*b*) An enlarged view of some diffraction spots is shown with Miller indices.

A typical diffraction image is shown in Fig. 3 ▶. The estimated mosaicity is 3.9°, which is larger than that observed for normal single crystals on the same instrument (around 0.8°).

**Figure 4:**
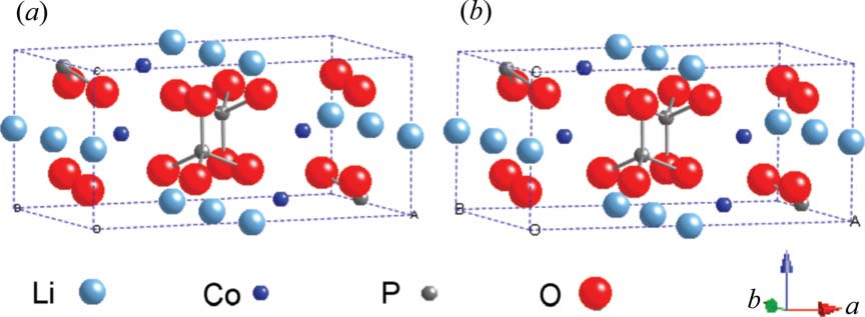
Comparison of the crystal structures of LiCoPO_4_ (*a*) determined using a pseudo single crystal prepared during the present study and (*b*) as reported in the literature (Kubel, 1994 ▶) using a traditional single crystal.

The cell constants and an orientation matrix for data collection corresponded to a primitive orthorhombic cell with dimensions *a* = 10.202 (6), *b* = 5.918 (3), *c* = 4.709 (2) Å, *V* = 284.3 (3) Å^3^ and *Z* = 4. The space group was determined to be *Pnma* (No. 62). These results are the same as those reported by Kubel (1994 ▶), who used a 0.090 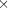 0.108 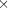 0.158 mm single crystal. The structure was solved by direct methods and expanded using Fourier techniques. The graphical display shown in Fig. 4 ▶ compares the present result with that from the literature, showing that the atomic coordinates determined in this study (see supplementary material^1^ for crystallographic data) are in excellent agreement with those determined using a traditional single crystal. The *R*1 and *wR*2 values were 6.59 and 16.8%, respectively.

The pseudo-single-crystal method (three-dimensional orientation) is applicable to the biaxial crystal systems (triclinic, monoclinic and orthorhombic systems) for which three susceptibilities are different. Uniaxial crystal systems (trigonal, hexagonal and tetragonal systems), for which two out of three susceptibilities are equal, undergo only uniaxial alignment, resulting in a fibre diffraction pattern. An isotropic crystal system (cubic system) does not align under any magnetic fields because its three susceptibilities are all equal.

## 4. Conclusions

In conclusion, it was demonstrated that it is possible to solve the crystal structure using a PSC prepared from a powder sample. In this study, crystalline microrods of LiCoPO_4_ were used for the purpose of demonstration. A powder sample suspended in a UV-curable monomer was aligned three-dimensionally under a dynamic elliptical magnetic field and the alignment achieved was consolidated by UV irradiation. The crystal structure was successfully indexed and solved using the standard method of single-crystal X-ray analysis, and it was in excellent agreement with the crystal structure reported previously. The technique presented here provides a third approach (the PSC method) for crystal structure analysis, after the single-crystal and powder methods.

## Supplementary Material

Crystal structure: contains datablocks General, toritsu_3T_2nd. DOI: 10.1107/S0021889809013430/db5060sup1.cif

Structure factors: contains datablocks toritsu_3T_2nd. DOI: 10.1107/S0021889809013430/db5060sup2.hkl

Supplementary material file. DOI: 10.1107/S0021889809013430/db5060sup3.pdf

## Footnotes

1 Supplementary material is available from the IUCr electronic archives (Reference: DB5060). Services for accessing this material are described at the back of the journal.
