# Supplementary material for: The pseudo-single-crystal method: a third approach to crystal structure determination
Source: J Appl Crystallogr. 2009 May 15;42(Pt 3):535–7. doi: 10.1107/S0021889809013430 (PMC3246822; doi:10.1107/S0021889809013430)
Supplement: Supplementary file 3 [file j-42-00535-sup3.pdf]

--- SUPPORTING INFORMATION ---

The pseudo-single crystal method: a third approach to crystal structure determination

Tsunehisa Kimura,[a]* Chengkang Chang,[b,†] Fumiko Kimura[b,§] and Masataka Maeyama[c]

[a]Graduate School of Agriculture, Kyoto University, Kitashirakawa, Sakyo-ku, Kyoto 606-8502, Japan, [b]Tsukuba Magnet Laboratory, National Institute for Materials Science, 3-13 Sakura, Tsukuba, Ibaraki 305-0003, Japan, [c]Rigaku Corporation, 3-9-12 Matsubara-cho, Akishima, Tokyo 196-8666, Japan. Correspondence e-mail: tkimura@kais.kyoto-u.ac.jp

Present address: [†]School of Materials Science and Engineering, Shanghai Jiaotong University, [§]Graduate School of Agriculture, Kyoto University

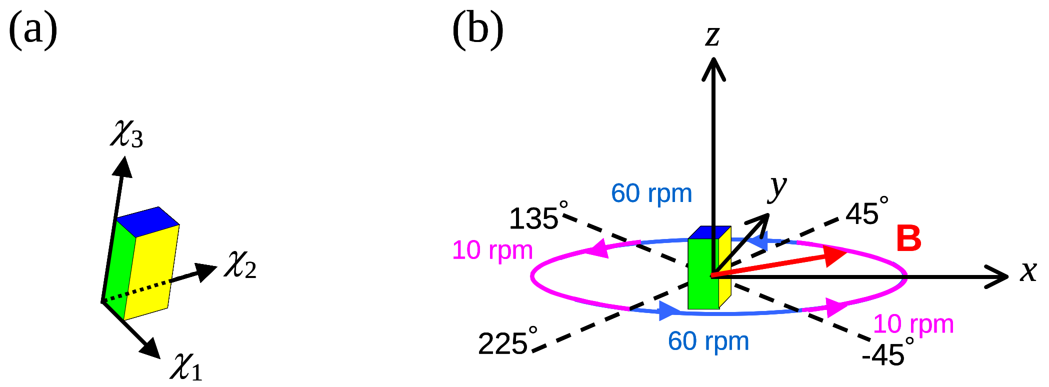

Figure S1. Schematic diagram showing the magnetic orientation of a biaxial crystal in a frequency modulated elliptical magnetic field. (a) A biaxial crystal has three different magnetic susceptibility axes, $\chi_3 < \chi_2 < \chi_1$. The largest one χ_1 and the smallest one χ_3 are referred to as easy and hard axes, respectively. (b) By application of a frequency-modulated elliptical magnetic field *B*, χ_1 aligns parallel to the *x*-axis and χ_3 aligns parallel to the *z*-axis. The rotation speed ω should be fast enough so that $\omega\tau >> 1$, where τ is the time required for orientation in a static field. In this explanation, the magnetic field is assumed to rotate non-uniformaly on the *xy*-plane, but in the actual experiment the sample is rotated non-uniformly in a static magnetic field, resulting in the same effect.

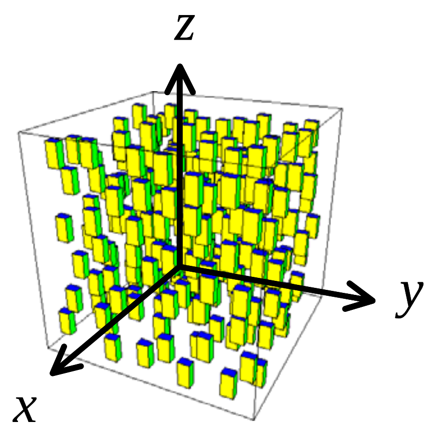

Figure S2. Schematic picture of a pseudo-single crystal (PSC). All crystallites are aligned in the same direction three- dimensionally in a matrix resin, giving rise to XRD equivalent to a single crystal.

X-ray Structure Report

Experimental

Data Collection

A violet platelet crystal of $LiCoPO_4$ having approximate dimensions of 3.00 x 3.00 x 0.30 mm was mounted on a glass fiber. All measurements were made on a Rigaku RAXIS RAPID imaging plate area detector with graphite monochromated Mo-Kα radiation.

Indexing was performed from 3 oscillations that were exposed for 30 seconds. The crystal-to-detector distance was 127.40 mm.

Cell constants and an orientation matrix for data collection corresponded to a primitive orthorhombic cell with dimensions:

a = 10.202(6) Å

b = 5.918(3) Å

c = 4.709(2) Å

V = 284.3(3) $Å^3$

For Z = 4 and F.W. = 160.85, the calculated density is 3.758 g/cm^3. Based on the systematic absences of:

0kl: k+l ± 2n

hk0: h ± 2n

packing considerations, a statistical analysis of intensity distribution, and the successful solution and refinement of the structure, the space group was determined to be:

The data were collected at a temperature of -30 $\pm$ 1°C to a maximum 2θ value of 55.0^{o}. A total of 96 oscillation images were collected. A sweep of data was done using ω scans from 130.0 to 190.0^{o} in 5.0^{o} step, at χ=45.0^{o} and ϕ = 0.0^{o}. The exposure rate was 60.0 [sec./^{o}]. A second sweep was performed using ω scans from 0.0 to 160.0^{o} in 5.0^{o} step, at χ=45.0^{o} and ϕ = 180.0^{o}. The exposure rate was 60.0 [sec./^{o}]. Another sweep was performed using ω scans from 0.0 to 260.0^{o} in 5.0^{o} step, at χ=0.0^{o} and ϕ = 0.0^{o}. The exposure rate was 60.0 [sec./^{o}]. The crystal-to-detector distance was 127.40 mm. Readout was performed in the 0.100 mm pixel mode.

Data Reduction

Of the 4825 reflections that were collected, 360 were unique (R_{int} = 0.133); equivalent reflections were merged.

The linear absorption coefficient, μ, for Mo-Kα radiation is 64.071 cm^{-1}. An empirical absorption correction was applied which resulted in transmission factors ranging from 0.030 to 0.146. The data were corrected for Lorentz and polarization effects.

Structure Solution and Refinement

The structure was solved by direct methods[1] and expanded using Fourier techniques[2]. The non-hydrogen atoms were refined anisotropically. The final cycle of full-matrix least-squares refinement[3] on F^2 was based on 345 observed reflections and 41 variable parameters and converged (largest parameter shift was 0.00 times its esd) with unweighted and weighted agreement factors of:

$$R1 = \Sigma ||Fo| - |Fc|| / \Sigma |Fo| = 0.0659$$

$$wR2 = [\Sigma (w (Fo^2 - Fc^2)^2)/ \Sigma w(Fo^2)^2]^{1/2} = 0.1680$$

The standard deviation of an observation of unit weight[4] was 1.12. A Sheldrick weighting scheme was used. The maximum and minimum peaks on the final difference Fourier map corresponded to 1.44 and -1.13 $e^-/Å^3$, respectively.

Neutral atom scattering factors were taken from Cromer and Waber[5]. Anomalous dispersion effects were included in Fcalc[6]; the values for $\Delta f'$ and $\Delta f''$ were those of Creagh and McAuley[7]. The values for the mass attenuation coefficients are those of Creagh and Hubbell[8]. All calculations were performed using the CrystalStructure[9] crystallographic software package except for refinement, which was performed using SHELXL-97[10].

References

(1) SIR97: Altomare, A., Burla, M., Camalli, M., Cascarano, G., Giacovazzo, C., Guagliardi, A., Moliterni, A., Polidori, G., and Spagna, R. (1999). J. Appl. Cryst., 32, 115-119.

(2) DIRDIF99: Beurskens, P.T., Admiraal, G., Beurskens, G., Bosman, W.P., de Gelder, R., Israel, R. and Smits, J.M.M.(1999). The DIRDIF-99 program system, Technical Report of the Crystallography Laboratory, University of Nijmegen, The Netherlands.

(3) Least Squares function minimized: (SHELXL97)

$$\Sigma w(F_o^2-F_c^2)^2 \quad \text{where w = Least Squares weights.}$$

(4) Standard deviation of an observation of unit weight:

$$[\Sigma w(F_o^2-F_c^2)^2/(N_o-N_v)]^{1/2}$$

where: N_o = number of observations

N_V = number of variables

(5) Cromer, D. T. & Waber, J. T.; "International Tables for X-ray Crystallography", Vol. IV, The Kynoch Press, Birmingham, England, Table 2.2 A (1974).

(6) Ibers, J. A. & Hamilton, W. C.; Acta Crystallogr., 17, 781 (1964).

(7) Creagh, D. C. & McAuley, W.J .; "International Tables for Crystallography", Vol C, (A.J.C. Wilson, ed.), Kluwer Academic Publishers, Boston, Table 4.2.6.8, pages 219-222 (1992).

(8) Creagh, D. C. & Hubbell, J.H..; "International Tables for Crystallography", Vol C, (A.J.C. Wilson, ed.), Kluwer Academic Publishers, Boston, Table 4.2.4.3, pages 200-206 (1992).

(9) CrystalStructure 3.8: Crystal Structure Analysis Package, Rigaku and Rigaku/MSC (2000-2006). 9009 New Trails Dr. The Woodlands TX 77381 USA.

(10) SHELX97: Sheldrick, G.M. (1997).

A. Crystal Data

Empirical Formula	$LiCoPO_4$
Formula Weight	160.85
Crystal Color, Habit	violet, platelet
Crystal Dimensions	3.00 X 3.00 X 0.30 mm
Crystal System	orthorhombic
Lattice Type	Primitive
Indexing Images	3 oscillations @ 30.0 seconds
Detector Position	127.40 mm
Pixel Size	0.100 mm
Lattice Parameters	a = 10.202(6) Å
	b = 5.918(3) Å

	c = 4.709(2) Å
	V = 284.3(3) $Å^3$
Space Group	Pnma (#62)
Z value	4
D_{calc}	3.758 g/cm^3
F_{000}	308.00
μ(MoKα)	64.071 cm^{-1}

B. Intensity Measurements

Diffractometer	Rigaku RAXIS-RAPID
Radiation	MoKα (λ = 0.71075 Å)<br>graphite monochromated
Detector Aperture	280 mm x 256 mm
Data Images	96 exposures
ω oscillation Range (χ=45.0, φ=0.0)	130.0 - 190.0^{o}
Exposure Rate	60.0 sec./^{o}
ω oscillation Range (χ=45.0, φ=180.0)	0.0 - 160.0^{o}
Exposure Rate	60.0 sec./^{o}
ω oscillation Range (χ=0.0, φ=0.0)	0.0 - 260.0^{o}
Exposure Rate	60.0 sec./^{o}
Detector Position	127.40 mm

Pixel Size	0.100 mm
$2\theta_{max}$	55.0^{o}
No. of Reflections Measured	Total: 4825
	Unique: 345 (R_{int} = 0.133)
Corrections	Lorentz-polarization
	Absorption
	(trans. factors: 0.030 - 0.146)

C. Structure Solution and Refinement

Structure Solution	Direct Methods (SIR97)
Refinement	Full-matrix least-squares on F^2
Function Minimized	Σ w $(Fo^2 - Fc^2)^2$
Least Squares Weights	$w = 1/ [\sigma^2(Fo^2) + (0.0479 \cdot P)^2 + 4.9483 \cdot P]$ where $P = (Max(Fo^2,0) + 2Fc^2)/3$
$2\theta_{max}$ cutoff	55.0^o
Anomalous Dispersion	All non-hydrogen atoms
No. Observations (All reflections)	345
No. Variables	41
Reflection/Parameter Ratio	8.41
Residuals: R1 (I>2.00σ(I))	0.0659
Residuals: R (All reflections)	0.0815
Residuals: wR2 (All reflections)	0.1680

Goodness of Fit Indicator	1.116
Max Shift/Error in Final Cycle	0.000
Maximum peak in Final Diff. Map	1.44 $e^-/Å^3$
Minimum peak in Final Diff. Map	-1.13 $e^-/Å^3$

Table 1. Atomic coordinates and B_{iso}/B_{eq} and occupancy

atom	x	y	z	B_{eq}	occ
Co(1)	0.72170(16)	0.2500	0.5213(4)	2.64(5)	1/2
P(1)	0.5940(3)	0.7500	0.5816(8)	2.64(6)	1/2
O(1)	0.6655(5)	0.5435(10)	0.7200(13)	2.88(11)	
O(2)	0.4522(8)	0.7500	0.704(2)	2.81(15)	1/2
O(3)	0.5982(8)	0.7500	0.257(2)	2.65(15)	1/2
Li(1)	0.5000	0.5000	1.0000	2.5(3)	1/2

$B_{eq} = 8/3\ \pi^2(U_{11}(aa^*)^2 + U_{22}(bb^*)^2 + U_{33}(cc^*)^2 + 2U_{12}(aa^*bb^*)\cos\gamma + 2U_{13}(aa^*cc^*)\cos\beta + 2U_{23}(bb^*cc^*)\cos\alpha)$

Table 2. Anisotropic displacement parameters

atom	U_{11}	U_{22}	U_{33}	U_{12}	U_{13}	U_{23}
Co(1)	0.0324(9)	0.0229(9)	0.0448(12)	0.0000	0.0004(6)	0.0000
P(1)	0.0305(15)	0.0234(15)	0.046(2)	0.0000	0.0012(13)	0.0000
O(1)	0.039(3)	0.027(3)	0.044(3)	0.004(2)	-0.007(2)	-0.000(2)
O(2)	0.033(4)	0.029(4)	0.045(5)	0.0000	0.002(3)	0.0000
O(3)	0.035(4)	0.016(3)	0.050(5)	0.0000	0.004(3)	0.0000
Li(1)	0.023(8)	0.020(8)	0.052(12)	-0.011(7)	0.006(7)	-0.002(8)

The general temperature factor expression: $\exp(-2\pi^2(a^{*2}U_{11}h^2 + b^{*2}U_{22}k^2 + c^{*2}U_{33}l^2 + 2a^*b^*U_{12}hk + 2a^*c^*U_{13}hl + 2b^*c^*U_{23}kl))$

Table 3. Bond lengths (Å)

atom	atom	distance	atom	atom	distance
Co(1)	O(1)	2.055(6)	Co(1)	O(1)$^{1)}$	2.198(6)
Co(1)	O(1)$^{2)}$	2.055(6)	Co(1)	O(1)$^{3)}$	2.198(6)
Co(1)	O(2)$^{4)}$	2.067(8)	Co(1)	O(3)$^{5)}$	2.146(8)
P(1)	O(1)	1.565(6)	P(1)	O(1)$^{6)}$	1.565(6)
P(1)	O(2)	1.557(8)	P(1)	O(3)	1.529(10)
P(1)	Li(1)	2.644(3)	P(1)	Li(1)$^{6)}$	2.644(3)
O(1)	Li(1)	2.157(6)	O(2)	Li(1)	2.090(6)
O(2)	Li(1)$^{6)}$	2.090(6)	O(3)	Li(1)$^{7)}$	2.158(6)
O(3)	Li(1)$^{8)}$	2.158(6)			

Symmetry Operators:

(1) -X+1/2+1,Y+1/2-1,Z+1/2-1 (2) X,-Y+1/2,Z

(3) -X+1/2+1,-Y+1,Z+1/2-1 (4) -X+1,-Y+1,-Z+1

(5) -X+1/2+1,Y+1/2-1,Z+1/2 (6) X,-Y+1/2+1,Z

(7) X,Y,Z-1 (8) X,-Y+1/2+1,Z-1

Table 4. Bond angles (^{o})

atom	atom	atom	angle	atom	atom	atom	angle
O(1)	Co(1)	$O(1)^{1)}$	155.5(2)	O(1)	Co(1)	$O(1)^{2)}$	115.4(2)
O(1)	Co(1)	$O(1)^{3)}$	88.3(2)	O(1)	Co(1)	$O(2)^{4)}$	89.7(2)
O(1)	Co(1)	$O(3)^{5)}$	90.2(2)	$O(1)^{1)}$	Co(1)	$O(1)^{2)}$	88.3(2)
$O(1)^{1)}$	Co(1)	$O(1)^{3)}$	67.6(2)	$O(1)^{1)}$	Co(1)	$O(2)^{4)}$	96.8(2)
$O(1)^{1)}$	Co(1)	$O(3)^{5)}$	83.4(2)	$O(1)^{2)}$	Co(1)	$O(1)^{3)}$	155.5(2)
$O(1)^{2)}$	Co(1)	$O(2)^{4)}$	89.7(2)	$O(1)^{2)}$	Co(1)	$O(3)^{5)}$	90.2(2)
$O(1)^{3)}$	Co(1)	$O(2)^{4)}$	96.8(2)	$O(1)^{3)}$	Co(1)	$O(3)^{5)}$	83.4(2)
$O(2)^{4)}$	Co(1)	$O(3)^{5)}$	179.7(3)	O(1)	P(1)	$O(1)^{6)}$	102.7(3)
O(1)	P(1)	O(2)	106.2(3)	O(1)	P(1)	O(3)	113.8(3)
O(1)	P(1)	Li(1)	54.7(2)	O(1)	P(1)	$Li(1)^{6)}$	107.2(2)
$O(1)^{6)}$	P(1)	O(2)	106.2(3)	$O(1)^{6)}$	P(1)	O(3)	113.8(3)
$O(1)^{6)}$	P(1)	Li(1)	107.2(2)	$O(1)^{6)}$	P(1)	$Li(1)^{6)}$	54.7(2)
O(2)	P(1)	O(3)	113.3(5)	O(2)	P(1)	Li(1)	52.2(2)
O(2)	P(1)	$Li(1)^{6)}$	52.2(2)	O(3)	P(1)	Li(1)	139.03(18)
O(3)	P(1)	$Li(1)^{6)}$	139.03(18)	Li(1)	P(1)	$Li(1)^{6)}$	68.06(8)
Co(1)	O(1)	$Co(1)^{7)}$	128.2(2)	Co(1)	O(1)	P(1)	126.9(3)
Co(1)	O(1)	Li(1)	113.3(2)	$Co(1)^{7)}$	O(1)	P(1)	94.5(2)
$Co(1)^{7)}$	O(1)	Li(1)	94.7(2)	P(1)	O(1)	Li(1)	89.0(2)
$Co(1)^{4)}$	O(2)	P(1)	127.4(5)	$Co(1)^{4)}$	O(2)	Li(1)	122.8(2)
$Co(1)^{4)}$	O(2)	$Li(1)^{6)}$	122.8(2)	P(1)	O(2)	Li(1)	91.7(3)
P(1)	O(2)	$Li(1)^{6)}$	91.7(3)	Li(1)	O(2)	$Li(1)^{6)}$	90.1(3)
$Co(1)^{8)}$	O(3)	P(1)	122.7(4)	$Co(1)^{8)}$	O(3)	$Li(1)^{9)}$	96.2(3)
$Co(1)^{8)}$	O(3)	$Li(1)^{10)}$	96.2(3)	P(1)	O(3)	$Li(1)^{9)}$	123.2(3)
P(1)	O(3)	$Li(1)^{10)}$	123.2(3)	$Li(1)^{9)}$	O(3)	$Li(1)^{10)}$	86.6(3)
P(1)	Li(1)	$P(1)^{11)}$	0(359)	P(1)	Li(1)	O(1)	36.30(16)

P(1)	Li(1)	O(1)$^{11)}$	143.70(16)	P(1)	Li(1)	O(2)	36.1(2)
P(1)	Li(1)	O(2)$^{11)}$	143.9(2)	P(1)	Li(1)	O(3)$^{12)}$	82.3(2)
P(1)	Li(1)	O(3)$^{4)}$	97.7(2)	P(1)$^{11)}$	Li(1)	O(1)	143.70(16)
P(1)$^{11)}$	Li(1)	O(1)$^{11)}$	36.30(16)	P(1)$^{11)}$	Li(1)	O(2)	143.9(2)
P(1)$^{11)}$	Li(1)	O(2)$^{11)}$	36.1(2)	P(1)$^{11)}$	Li(1)	O(3)$^{12)}$	97.7(2)
P(1)$^{11)}$	Li(1)	O(3)$^{4)}$	82.3(2)	O(1)	Li(1)	O(1)$^{11)}$	180.0(3)
O(1)	Li(1)	O(2)	72.0(2)	O(1)	Li(1)	O(2)$^{11)}$	108.0(2)
O(1)	Li(1)	O(3)$^{12)}$	84.1(2)	O(1)	Li(1)	O(3)$^{4)}$	95.9(2)
O(1)$^{11)}$	Li(1)	O(2)	108.0(2)	O(1)$^{11)}$	Li(1)	O(2)$^{11)}$	72.0(2)
O(1)$^{11)}$	Li(1)	O(3)$^{12)}$	95.9(2)	O(1)$^{11)}$	Li(1)	O(3)$^{4)}$	84.1(2)
O(2)	Li(1)	O(2)$^{11)}$	0(359)	O(2)	Li(1)	O(3)$^{12)}$	89.8(2)
O(2)	Li(1)	O(3)$^{4)}$	90.2(2)	O(2)$^{11)}$	Li(1)	O(3)$^{12)}$	90.2(2)

Table 4. Bond angles (^{o}) (continued)

atom	atom	atom	angle	atom	atom	atom	angle
O(2)$^{11)}$	Li(1)	O(3)$^{4)}$	89.8(2)	O(3)$^{12)}$	Li(1)	O(3)$^{4)}$	180.0(3)

Symmetry Operators:

(1) -X+1/2+1,Y+1/2-1,Z+1/2-1

(2) X,-Y+1/2,Z

(3) -X+1/2+1,-Y+1,Z+1/2-1

(4) -X+1,-Y+1,-Z+1

(5) -X+1/2+1,Y+1/2-1,Z+1/2

(6) X,-Y+1/2+1,Z

(7) -X+1/2+1,Y+1/2,Z+1/2

(8) -X+1/2+1,Y+1/2,Z+1/2-1

(9) X,Y,Z-1

(10) X,-Y+1/2+1,Z-1

(11) -X+1,-Y+1,-Z+2

(12) X,Y,Z+1

Table 5. Torsion Angles(^{o})

atom1	atom2	atom3	atom4	angle	atom1	atom2	atom3	atom4	angle
O(1)	Co(1)	O(1)$^{1)}$	Co(1)$^{1)}$	-149.9(4)	O(1)	Co(1)	O(1)$^{1)}$	P(1)$^{1)}$	-4.0(7)
O(1)	Co(1)	O(1)$^{1)}$	Li(1)$^{1)}$	85.4(6)	O(1)$^{1)}$	Co(1)	O(1)	Co(1)$^{2)}$	-70.1(7)
O(1)$^{1)}$	Co(1)	O(1)	P(1)	65.5(7)	O(1)$^{1)}$	Co(1)	O(1)	Li(1)	173.0(4)
O(1)	Co(1)	O(1)$^{3)}$	Li(1)$^{3)}$	22.2(3)	O(1)$^{3)}$	Co(1)	O(1)	Co(1)$^{2)}$	94.6(4)
O(1)$^{3)}$	Co(1)	O(1)	P(1)	-129.8(4)	O(1)$^{3)}$	Co(1)	O(1)	Li(1)	-22.2(3)
O(1)	Co(1)	O(1)$^{4)}$	Li(1)$^{4)}$	80.9(2)	O(1)$^{4)}$	Co(1)	O(1)	Co(1)$^{2)}$	-79.1(3)
O(1)$^{4)}$	Co(1)	O(1)	P(1)	56.5(4)	O(1)$^{4)}$	Co(1)	O(1)	Li(1)	164.0(3)
O(1)	Co(1)	O(2)$^{5)}$	P(1)$^{5)}$	-57.71(17)	O(2)$^{5)}$	Co(1)	O(1)	Co(1)$^{2)}$	-175.9(4)
O(2)$^{5)}$	Co(1)	O(1)	P(1)	-40.3(4)	O(2)$^{5)}$	Co(1)	O(1)	Li(1)	67.2(3)
O(1)	Co(1)	O(3)$^{6)}$	P(1)$^{6)}$	57.71(17)	O(3)$^{6)}$	Co(1)	O(1)	Co(1)$^{2)}$	4.3(4)
O(3)$^{6)}$	Co(1)	O(1)	P(1)	139.9(4)	O(3)$^{6)}$	Co(1)	O(1)	Li(1)	-112.6(3)
O(1)$^{1)}$	Co(1)	O(1)$^{3)}$	Li(1)$^{3)}$	-164.0(3)	O(1)$^{3)}$	Co(1)	O(1)$^{1)}$	Co(1)$^{1)}$	43.9(3)
O(1)$^{3)}$	Co(1)	O(1)$^{1)}$	P(1)$^{1)}$	-170.3(3)	O(1)$^{3)}$	Co(1)	O(1)$^{1)}$	Li(1)$^{1)}$	-80.9(2)
O(1)$^{1)}$	Co(1)	O(1)$^{4)}$	Li(1)$^{4)}$	-95.1(2)	O(1)$^{4)}$	Co(1)	O(1)$^{1)}$	Co(1)$^{1)}$	-140.2(4)
O(1)$^{4)}$	Co(1)	O(1)$^{1)}$	P(1)$^{1)}$	5.7(2)	O(1)$^{4)}$	Co(1)	O(1)$^{1)}$	Li(1)$^{1)}$	95.1(2)
O(1)$^{1)}$	Co(1)	O(2)$^{5)}$	P(1)$^{5)}$	145.94(16)	O(2)$^{5)}$	Co(1)	O(1)$^{1)}$	Co(1)$^{1)}$	-45.6(3)
O(2)$^{5)}$	Co(1)	O(1)$^{1)}$	P(1)$^{1)}$	100.3(3)	O(2)$^{5)}$	Co(1)	O(1)$^{1)}$	Li(1)$^{1)}$	-170.3(2)
O(1)$^{1)}$	Co(1)	O(3)$^{6)}$	P(1)$^{6)}$	-145.96(16)	O(3)$^{6)}$	Co(1)	O(1)$^{1)}$	Co(1)$^{1)}$	134.3(3)
O(3)$^{6)}$	Co(1)	O(1)$^{1)}$	P(1)$^{1)}$	-79.9(3)	O(3)$^{6)}$	Co(1)	O(1)$^{1)}$	Li(1)$^{1)}$	9.5(2)
O(1)$^{3)}$	Co(1)	O(1)$^{4)}$	Li(1)$^{4)}$	-85.4(6)	O(1)$^{4)}$	Co(1)	O(1)$^{3)}$	Li(1)$^{3)}$	-173.0(4)
O(1)$^{3)}$	Co(1)	O(2)$^{5)}$	P(1)$^{5)}$	57.71(17)	O(2)$^{5)}$	Co(1)	O(1)$^{3)}$	Li(1)$^{3)}$	-67.2(3)
O(1)$^{3)}$	Co(1)	O(3)$^{6)}$	P(1)$^{6)}$	-57.71(17)	O(3)$^{6)}$	Co(1)	O(1)$^{3)}$	Li(1)$^{3)}$	112.6(3)
O(1)$^{4)}$	Co(1)	O(2)$^{5)}$	P(1)$^{5)}$	-145.94(16)	O(2)$^{5)}$	Co(1)	O(1)$^{4)}$	Li(1)$^{4)}$	170.3(2)
O(1)$^{4)}$	Co(1)	O(3)$^{6)}$	P(1)$^{6)}$	145.96(16)	O(3)$^{6)}$	Co(1)	O(1)$^{4)}$	Li(1)$^{4)}$	-9.5(2)
O(1)	P(1)	O(1)$^{7)}$	Co(1)$^{2)}$	-7.6(3)	O(1)	P(1)	O(1)$^{7)}$	Li(1)$^{7)}$	-102.2(3)

O(1)$^{7)}$	P(1)	O(1)	Co(1)	-138.9(4)	O(1)$^{7)}$	P(1)	O(1)	Co(1)$^{2)}$	7.6(3)
O(1)$^{7)}$	P(1)	O(1)	Li(1)	102.2(3)	O(1)	P(1)	O(2)	Co(1)$^{5)}$	-125.6(2)
O(1)	P(1)	O(2)	Li(1)	9.3(3)	O(1)	P(1)	O(2)	Li(1)$^{7)}$	99.5(2)
O(2)	P(1)	O(1)	Co(1)	109.8(4)	O(2)	P(1)	O(1)	Co(1)$^{2)}$	-103.6(3)
O(2)	P(1)	O(1)	Li(1)	-9.0(3)	O(1)	P(1)	O(3)	Co(1)$^{8)}$	-58.6(3)
O(1)	P(1)	O(3)	Li(1)$^{9)}$	66.4(5)	O(1)	P(1)	O(3)	Li(1)$^{10)}$	176.4(3)
O(3)	P(1)	O(1)	Co(1)	-15.5(5)	O(3)	P(1)	O(1)	Co(1)$^{2)}$	131.0(3)
O(3)	P(1)	O(1)	Li(1)	-134.4(3)	O(1)	P(1)	Li(1)	O(2)	-169.0(4)
O(1)	P(1)	Li(1)	O(2)$^{11)}$	11.0(4)	O(1)	P(1)	Li(1)	O(3)$^{12)}$	90.6(3)
O(1)	P(1)	Li(1)	O(3)$^{5)}$	-89.4(3)	Li(1)	P(1)	O(1)	Co(1)	118.9(4)
Li(1)	P(1)	O(1)	Co(1)$^{2)}$	-94.6(2)	O(1)	P(1)	Li(1)$^{7)}$	O(1)$^{7)}$	93.6(3)
O(1)	P(1)	Li(1)$^{7)}$	O(2)	-97.4(3)	O(1)	P(1)	Li(1)$^{7)}$	O(3)$^{12)}$	3.0(3)

Table 5. Torsion angles (°) (continued)

atom1	atom2	atom3	atom4	angle	atom1	atom2	atom3	atom4	angle
Li(1)$^{7)}$	P(1)	O(1)	Co(1)	164.5(3)	Li(1)$^{7)}$	P(1)	O(1)	Co(1)$^{2)}$	-49.0(2)
Li(1)$^{7)}$	P(1)	O(1)	Li(1)	45.7(2)	O(1)$^{7)}$	P(1)	O(2)	Co(1)$^{5)}$	125.6(2)
O(1)$^{7)}$	P(1)	O(2)	Li(1)	-99.5(2)	O(1)$^{7)}$	P(1)	O(2)	Li(1)$^{7)}$	-9.3(3)
O(2)	P(1)	O(1)$^{7)}$	Co(1)$^{2)}$	103.6(3)	O(2)	P(1)	O(1)$^{7)}$	Li(1)$^{7)}$	9.0(3)
O(1)$^{7)}$	P(1)	O(3)	Co(1)$^{8)}$	58.6(3)	O(1)$^{7)}$	P(1)	O(3)	Li(1)$^{9)}$	-176.4(3)
O(1)$^{7)}$	P(1)	O(3)	Li(1)$^{10)}$	-66.4(5)	O(3)	P(1)	O(1)$^{7)}$	Co(1)$^{2)}$	-131.0(3)
O(3)	P(1)	O(1)$^{7)}$	Li(1)$^{7)}$	134.4(3)	O(1)$^{7)}$	P(1)	Li(1)	O(1)	-93.6(3)
O(1)$^{7)}$	P(1)	Li(1)	O(2)	97.4(3)	O(1)$^{7)}$	P(1)	Li(1)	O(1)$^{11)}$	86.4(3)
O(1)$^{7)}$	P(1)	Li(1)	O(2)$^{11)}$	-82.6(3)	O(1)$^{7)}$	P(1)	Li(1)	O(3)$^{12)}$	-3.0(3)
O(1)$^{7)}$	P(1)	Li(1)	O(3)$^{5)}$	177.0(3)	Li(1)	P(1)	O(1)$^{7)}$	Co(1)$^{2)}$	49.0(2)
Li(1)	P(1)	O(1)$^{7)}$	Li(1)$^{7)}$	-45.7(2)	O(1)$^{7)}$	P(1)	Li(1)$^{7)}$	O(2)	169.0(4)
O(1)$^{7)}$	P(1)	Li(1)$^{7)}$	O(3)$^{12)}$	-90.6(3)	Li(1)$^{7)}$	P(1)	O(1)$^{7)}$	Co(1)$^{2)}$	94.6(2)
O(2)	P(1)	O(3)	Li(1)$^{9)}$	-55.0(3)	O(2)	P(1)	O(3)	Li(1)$^{10)}$	55.0(3)
O(3)	P(1)	O(2)	Li(1)	134.92(18)	O(3)	P(1)	O(2)	Li(1)$^{7)}$	-134.92(18)
O(2)	P(1)	Li(1)	O(1)	169.0(4)	O(2)	P(1)	Li(1)	O(1)$^{11)}$	-11.0(4)
O(2)	P(1)	Li(1)	O(3)$^{12)}$	-100.4(3)	O(2)	P(1)	Li(1)	O(3)$^{5)}$	79.6(3)
Li(1)	P(1)	O(2)	Co(1)$^{5)}$	-134.92(18)	Li(1)	P(1)	O(2)	Li(1)$^{7)}$	90.2(3)
O(2)	P(1)	Li(1)$^{7)}$	O(1)$^{7)}$	-169.0(4)	O(2)	P(1)	Li(1)$^{7)}$	O(3)$^{12)}$	100.4(3)
Li(1)$^{7)}$	P(1)	O(2)	Co(1)$^{5)}$	134.92(18)	Li(1)$^{7)}$	P(1)	O(2)	Li(1)	-90.2(3)
O(3)	P(1)	Li(1)	O(1)	86.4(5)	O(3)	P(1)	Li(1)	O(2)	-82.6(5)
O(3)	P(1)	Li(1)	O(1)$^{11)}$	-93.6(5)	O(3)	P(1)	Li(1)	O(2)$^{11)}$	97.4(5)
O(3)	P(1)	Li(1)	O(3)$^{12)}$	177.0(4)	O(3)	P(1)	Li(1)	O(3)$^{5)}$	-3.0(4)
Li(1)	P(1)	O(3)	Co(1)$^{8)}$	-121.4(3)	Li(1)	P(1)	O(3)	Li(1)$^{9)}$	3.6(7)
Li(1)	P(1)	O(3)	Li(1)$^{10)}$	113.61(15)	O(3)	P(1)	Li(1)$^{7)}$	O(1)$^{7)}$	-86.4(5)
O(3)	P(1)	Li(1)$^{7)}$	O(2)	82.6(5)	O(3)	P(1)	Li(1)$^{7)}$	O(3)$^{12)}$	-177.0(4)

Li(1)[7)]	P(1)	O(3)	Co(1)[8)]	121.4(3)	Li(1)[7)]	P(1)	O(3)	Li(1)[9)]	-113.61(15)
Li(1)[7)]	P(1)	O(3)	Li(1)[10)]	-3.6(7)	Li(1)	P(1)	Li(1)[7)]	O(1)[7)]	132.6(3)
Li(1)	P(1)	Li(1)[7)]	O(2)	-58.4(3)	Li(1)	P(1)	Li(1)[7)]	O(3)[12)]	41.98(18)
Li(1)[7)]	P(1)	Li(1)	O(1)	-132.6(3)	Li(1)[7)]	P(1)	Li(1)	O(2)	58.4(3)
Li(1)[7)]	P(1)	Li(1)	O(1)[11)]	47.4(3)	Li(1)[7)]	P(1)	Li(1)	O(2)[11)]	-121.6(3)
Li(1)[7)]	P(1)	Li(1)	O(3)[12)]	-41.98(18)	Li(1)[7)]	P(1)	Li(1)	O(3)[5)]	138.02(18)
Co(1)	O(1)	Li(1)	P(1)	-130.3(4)	Co(1)	O(1)	Li(1)	P(1)[11)]	49.7(4)
Co(1)	O(1)	Li(1)	O(2)	-123.5(3)	Co(1)	O(1)	Li(1)	O(2)[11)]	56.5(3)
Co(1)	O(1)	Li(1)	O(3)[12)]	144.7(3)	Co(1)	O(1)	Li(1)	O(3)[5)]	-35.3(3)
Co(1)[2)]	O(1)	Li(1)	P(1)	94.4(3)	Co(1)[2)]	O(1)	Li(1)	P(1)[11)]	-85.6(3)
Co(1)[2)]	O(1)	Li(1)	O(2)	101.2(2)	Co(1)[2)]	O(1)	Li(1)	O(2)[11)]	-78.8(2)
Co(1)[2)]	O(1)	Li(1)	O(3)[12)]	9.5(2)	Co(1)[2)]	O(1)	Li(1)	O(3)[5)]	-170.5(2)

Table 5. Torsion angles (°) (continued)

atom1	atom2	atom3	atom4	angle	atom1	atom2	atom3	atom4	angle
P(1)	O(1)	Li(1)	O(2)	6.8(2)	P(1)	O(1)	Li(1)	O(2)$^{11)}$	-173.2(2)
P(1)	O(1)	Li(1)	O(3)$^{12)}$	-85.0(2)	P(1)	O(1)	Li(1)	O(3)$^{5)}$	95.0(2)
Co(1)$^{5)}$	O(2)	Li(1)	P(1)	138.0(5)	Co(1)$^{5)}$	O(2)	Li(1)	P(1)$^{11)}$	-42.0(5)
Co(1)$^{5)}$	O(2)	Li(1)	O(1)	131.1(4)	Co(1)$^{5)}$	O(2)	Li(1)	O(1)$^{11)}$	-48.9(4)
Co(1)$^{5)}$	O(2)	Li(1)	O(3)$^{12)}$	-145.0(4)	Co(1)$^{5)}$	O(2)	Li(1)	O(3)$^{5)}$	35.0(4)
Co(1)$^{5)}$	O(2)	Li(1)$^{7)}$	P(1)	-138.0(5)	Co(1)$^{5)}$	O(2)	Li(1)$^{7)}$	O(1)$^{7)}$	-131.1(4)
Co(1)$^{5)}$	O(2)	Li(1)$^{7)}$	O(3)$^{12)}$	145.0(4)	P(1)	O(2)	Li(1)	O(1)	-6.8(2)
P(1)	O(2)	Li(1)	O(1)$^{11)}$	173.2(2)	P(1)	O(2)	Li(1)	O(3)$^{12)}$	77.1(3)
P(1)	O(2)	Li(1)	O(3)$^{5)}$	-102.9(3)	P(1)	O(2)	Li(1)$^{7)}$	O(1)$^{7)}$	6.8(2)
P(1)	O(2)	Li(1)$^{7)}$	O(3)$^{12)}$	-77.1(3)	Li(1)	O(2)	Li(1)$^{7)}$	P(1)	91.7(3)
Li(1)	O(2)	Li(1)$^{7)}$	O(1)$^{7)}$	98.5(2)	Li(1)	O(2)	Li(1)$^{7)}$	O(3)$^{12)}$	14.7(3)
Li(1)$^{7)}$	O(2)	Li(1)	P(1)	-91.7(3)	Li(1)$^{7)}$	O(2)	Li(1)	P(1)$^{11)}$	88.3(3)
Li(1)$^{7)}$	O(2)	Li(1)	O(1)	-98.5(2)	Li(1)$^{7)}$	O(2)	Li(1)	O(1)$^{11)}$	81.5(2)
Li(1)$^{7)}$	O(2)	Li(1)	O(3)$^{12)}$	-14.7(3)	Li(1)$^{7)}$	O(2)	Li(1)	O(3)$^{5)}$	165.3(3)
Co(1)$^{8)}$	O(3)	Li(1)$^{9)}$	P(1)$^{5)}$	133.75(18)	Co(1)$^{8)}$	O(3)	Li(1)$^{9)}$	P(1)$^{9)}$	-46.25(18)
Co(1)$^{8)}$	O(3)	Li(1)$^{9)}$	O(1)$^{9)}$	-9.7(2)	Co(1)$^{8)}$	O(3)	Li(1)$^{9)}$	O(2)$^{9)}$	-81.6(2)
Co(1)$^{8)}$	O(3)	Li(1)$^{9)}$	O(1)$^{5)}$	170.3(2)	Co(1)$^{8)}$	O(3)	Li(1)$^{9)}$	O(2)$^{5)}$	98.4(2)
Co(1)$^{8)}$	O(3)	Li(1)$^{10)}$	O(1)$^{10)}$	9.7(2)	P(1)	O(3)	Li(1)$^{9)}$	P(1)$^{5)}$	-2.4(4)
P(1)	O(3)	Li(1)$^{9)}$	P(1)$^{9)}$	177.6(4)	P(1)	O(3)	Li(1)$^{9)}$	O(1)$^{9)}$	-145.8(4)
P(1)	O(3)	Li(1)$^{9)}$	O(2)$^{9)}$	142.3(4)	P(1)	O(3)	Li(1)$^{9)}$	O(1)$^{5)}$	34.2(4)
P(1)	O(3)	Li(1)$^{9)}$	O(2)$^{5)}$	-37.7(4)	P(1)	O(3)	Li(1)$^{10)}$	O(1)$^{10)}$	145.8(4)
Li(1)$^{9)}$	O(3)	Li(1)$^{10)}$	O(1)$^{10)}$	-86.1(2)	Li(1)$^{10)}$	O(3)	Li(1)$^{9)}$	P(1)$^{5)}$	-130.4(2)
Li(1)$^{10)}$	O(3)	Li(1)$^{9)}$	P(1)$^{9)}$	49.6(2)	Li(1)$^{10)}$	O(3)	Li(1)$^{9)}$	O(1)$^{9)}$	86.1(2)
Li(1)$^{10)}$	O(3)	Li(1)$^{9)}$	O(2)$^{9)}$	14.2(3)	Li(1)$^{10)}$	O(3)	Li(1)$^{9)}$	O(1)$^{5)}$	-93.9(2)
Li(1)$^{10)}$	O(3)	Li(1)$^{9)}$	O(2)$^{5)}$	-165.8(3)					

Symmetry Operators:

(1) -X+1/2+1,Y+1/2-1,Z+1/2-1 (2) -X+1/2+1,Y+1/2,Z+1/2

(3) X,-Y+1/2,Z (4) -X+1/2+1,-Y+1,Z+1/2-1

(5) -X+1,-Y+1,-Z+1 (6) -X+1/2+1,Y+1/2-1,Z+1/2

(7) X,-Y+1/2+1,Z (8) -X+1/2+1,Y+1/2,Z+1/2-1

(9) X,Y,Z-1 (10) X,-Y+1/2+1,Z-1

(11) -X+1,-Y+1,-Z+2 (12) X,Y,Z+1

The sign is positive if when looking from atom 2 to atom 3 a clock-wise motion of atom 1 would superimpose it on atom 4.

Table 6. Distances beyond the asymmetric unit out to 3.60 Å

atom	atom	distance	atom	atom	distance
Co(1)	P(1)$^{1)}$	3.2457(14)	Co(1)	O(3)$^{1)}$	3.449(4)
P(1)	Co(1)$^{2)}$	3.2457(14)	P(1)	O(1)$^{3)}$	3.470(6)
P(1)	O(2)$^{4)}$	3.284(4)	O(1)	O(1)$^{5)}$	2.964(8)
O(1)	O(2)$^{6)}$	3.190(9)	O(2)	P(1)$^{4)}$	3.284(4)
O(2)	O(1)$^{7)}$	3.190(9)	O(2)	O(1)$^{3)}$	2.906(9)
O(2)	O(1)$^{8)}$	3.437(10)	O(2)	O(1)$^{9)}$	3.190(9)
O(2)	O(3)$^{4)}$	3.009(2)	O(3)	Co(1)$^{2)}$	3.449(4)
O(3)	O(1)$^{3)}$	3.204(9)	O(3)	O(2)$^{4)}$	3.009(2)
Li(1)	Co(1)$^{10)}$	3.2031(14)	Li(1)	O(1)$^{5)}$	3.576(5)
Li(1)	O(1)$^{11)}$	3.448(6)	Li(1)	O(1)$^{9)}$	3.576(5)

Symmetry Operators:

(1) X,Y-1,Z
(2) X,Y+1,Z
(3) -X+1,Y+1/2,-Z+1
(4) -X+1,-Y+2,-Z+1
(5) -X+1/2+1,-Y+1,Z+1/2
(6) X+1/2,-Y+1/2+1,-Z+1/2+1
(7) X+1/2-1,-Y+1/2+1,-Z+1/2+1
(8) -X+1,Y+1/2,-Z+2
(9) X+1/2-1,Y,-Z+1/2+1
(10) X+1/2-1,-Y+1/2,-Z+1/2+1
(11) -X+1,Y+1/2-1,-Z+2
